# The health policy response to COVID-19 in Malawi

**DOI:** 10.1136/bmjgh-2021-006035

**Published:** 2021-05-18

**Authors:** Grace W Mzumara, Marlen Chawani, Melody Sakala, Lily Mwandira, Elias Phiri, Edith Milanzi, Mphatso Dennis Phiri, Isabel Kazanga, Thomasena O’Byrne, Eliya M Zulu, Collins Mitambo, Titus Divala, Bertie Squire, Pui-Ying Iroh Tam

**Affiliations:** 1 Child Health, Malawi-Liverpool-Wellcome Trust Clinical Research Programme, Blantyre, Malawi; 2 Policy Engagement Unit, Malawi Liverpool Wellcome Trust Clinical Research Programme, Blantyre, Malawi; 3 African Institute for Development Policy, Lilongwe, Malawi; 4 Early life and Neonatal Infections, Malawi Liverpool Wellcome Trust Clinical Research Programme, Blantyre, Malawi; 5 MRC Clinical Trials Unit, University College London, London, UK; 6 Malawi-Liverpool-Wellcome Trust Clinical Research Programme, Blantyre, Blantyre, Malawi; 7 Health Economics and Policy Unit, University of Malawi College of Medicine, Lilongwe, Malawi; 8 Trinity College Dublin Trinity Centre for Global Health, Dublin, Ireland; 9 Department of Research, Malawi Ministry of Health, Lilongwe, Malawi; 10 Department of Infectious Disease Epidemiology, London School of Hygiene and Tropical Medicine, London, UK; 11 Clinical Sciences, Liverpool School of Tropical Medicine, Liverpool, UK; 12 Liverpool School of Tropical Medicine, Liverpool, UK

**Keywords:** COVID-19, health policy, health systems

## Abstract

Malawi declared a state of national disaster due to the COVID-19 pandemic on 20th March 2020 and registered its first confirmed coronavirus case on the 2 April 2020. The aim of this paper was to document policy decisions made in response to the COVID-19 pandemic from January to August 2020. We reviewed policy documents from the Public Health Institute of Malawi, the Malawi Gazette, the Malawi Ministry of Health and Population and the University of Oxford Coronavirus Government Response Tracker. We found that the Malawi response to the COVID-19 pandemic was multisectoral and implemented through 15 focused working groups termed clusters. Each cluster was charged with providing policy direction in their own area of focus. All clusters then fed into one central committee for major decisions and reporting to head of state. Key policies identified during the review include international travel ban, school closures at all levels, cancellation of public events, decongesting workplaces and public transport, and mandatory face coverings and a testing policy covering symptomatic people. Supportive interventions included risk communication and community engagement in multiple languages and over a variety of mediums, efforts to improve access to water, sanitation, nutrition and unconditional social-cash transfers for poor urban and rural households.

**Summary box**

- Malawi used a multisectoral approach to address health and socioeconomic aspects of the COVID-19 pandemic. This process successfully increased the COVID-19 testing capacity but, its complexities included resistance to some policies and unintended consequences.
- There was resistance to a stringent lockdown policy which demonstrated the need for epidemic response policies to be supported by public health laws and to mitigate both direct and indirect impacts of the COVID-19 pandemic in Malawi.
- Unintended consequences of the response policies included introducing remote learning to public education. A negative unintended consequence included an increase in teenage pregnancies during the school closure period.
- There is need to revise the Malawi public health act to reflect the current evidence on epidemic response, and to support frameworks that strengthen surveillance, health infrastructure and health information systems to manage the current and future epidemics.

## Introduction

Health policies inform decisions regarding population health and determine how they will be implemented, covering issues from primary healthcare to health emergencies.^1^ When WHO declared COVID-19 a pandemic on the 11 March 2020,^2^ various governments and agencies responded with a series of health policies to prevent the spread of the disease.

WHO published technical guidance aimed at reducing transmission, optimising clinical care and reducing the impact on health systems, social services and economic activity.^3^ Countries enacted policies to reduce the spread of the virus and manage the emerging disease and resulting socioeconomic burden. These involved implementing disease surveillance systems, disseminating public health information, enacting restrictions on gatherings and preparing healthcare facilities for clinical case management.^4^

In low/middle-income countries, the challenge was to manage the COVID-19 pandemic and pre-existing health priorities with weaker health systems than high income countries. For example, Divala *et al* noted that COVID-19 represents an increased mortality risk comparable to 4 months of background mortality among Malawians, compared with 12 months background mortality in the UK.^5^ This is because weak health systems in low-income countries may not have the capacity to manage the increased demands for healthcare. In this case, it is important for countries to form health policies that do not compromise their pre-existing quality of care and are appropriate to their socioeconomic circumstances.

This article documents Malawi’s health policy response to the COVID-19 pandemic from January 2020 to 31 August 2020, explores unintended consequences and suggests improvements to the health system. In this paper, health policy response to COVID-19 refers to decisions made by the Government of Malawi, to manage health and socio-economic impacts of the COVID-19 pandemic.

### Context

Malawi is a southern African country with a population of about 17.5 million, a median age of 17 years and average household size of 4.4.^6 7^ In 2019, 52.6% of Malawians had multidimensional poverty and 28.5% were vulnerable to poverty.^8^ In early 2020, Sonenthal *et al* found that out of 13 Malawian government hospitals, only four central hospitals were equipped to provide mechanical ventilation—with a total of 16 ventilators—and that oxygen was available in 10 general medical wards.^9^ These deficiencies in health infrastructure on a background of poverty and other pre-existing health priorities (eg, HIV and Tuberculosis) emphasise the importance of health policies to anticipate challenges and strategically manage the health and socioeconomic effects of the new pandemic. Without response policies to implement mitigation measures, mathematical modelling for the COVID-19 pandemic in Malawi predicted that 1.5 million infections and 50 000 deaths would occur as a direct result of the disease between March and June 2020.^10^

### Overview of COVID-19 in Malawi: January 2020 to August 2020

The first COVID-19 cases in Malawi were confirmed on the 2 April.^11^ These were three cases with one index case and two local transmission cases. As of the 31 August, there were 5566 confirmed cases and 175 deaths.^12^ Of the confirmed cases, the average age was 36 years and 66.9% were male.^12^ Among the confirmed COVID-19 deaths, the average age was 56.7 years and 82.5% were male.^12 13^ While initial cases were primarily imported, cases of local transmission (696 cases) first surpassed imported (640 cases), on the 1 July.^14^

### Approach

Our aim was to document key health policy decisions in response to the COVID-19 pandemic in Malawi January 2020 to 31 August 2020. We chose this period because we expect that all responses to the first wave of the pandemic should have occurred. We obtained information COVID-19 cases and deaths from the Malawi Ministry of Health COVID-19 dashboard.^15^

We reviewed documents from the Malawi National COVID-19 preparedness and response plan for May to December 2020, the Public Health Institute of Malawi (PHIM) updates on COVID-19 (available at: https: //malawipublichealth.org/) and the Malawi gazette to identify key policy decisions and announcements. We also used the University of Oxford Coronavirus Government Response Tracker (Available at: https://www.bsg.ox.ac.uk/research/research-projects/coronavirus-government-response-tracker) to identify containment, health and economic policies made by the government of Malawi.^16^ We used the Walt and Gilson policy triangle framework to describe the context, content, actors and process of policy formulation.^17^

## Findings

### The content of COVID-19 response policies

#### The COVID-19 response strategy

The response strategy was led by the MOHP and Department of Disaster Management Affairs.^18^ A multisectoral pandemic response was implemented using clusters to address direct health and indirect socioeconomic impacts of the pandemic.^18^ The clusters are multidisciplinary groups of experts and development partners that coordinate the response to health and socioeconomic impacts of the pandemic in Malawi. The 15 clusters were: public communication, health, water sanitation and hygiene, protection and social support, economic empowerment, employment and labour force protection, education, security and enforcement, food security, transport and logistics, agriculture, nutrition, local governance, shelter and camp management and intercluster coordination.^18^ Each cluster was led by various government departments and development partners which included the WHO, the World Food Programme, United Nations Children’s Fund, The Joint United Nations Programme on HIV and AIDS (UNAIDS), United Nations Development Programme and the United Nations Resident Coordinators Office.^18^ These partners were key to formulating and implementing policies within respective clusters.

**Figure 1:**
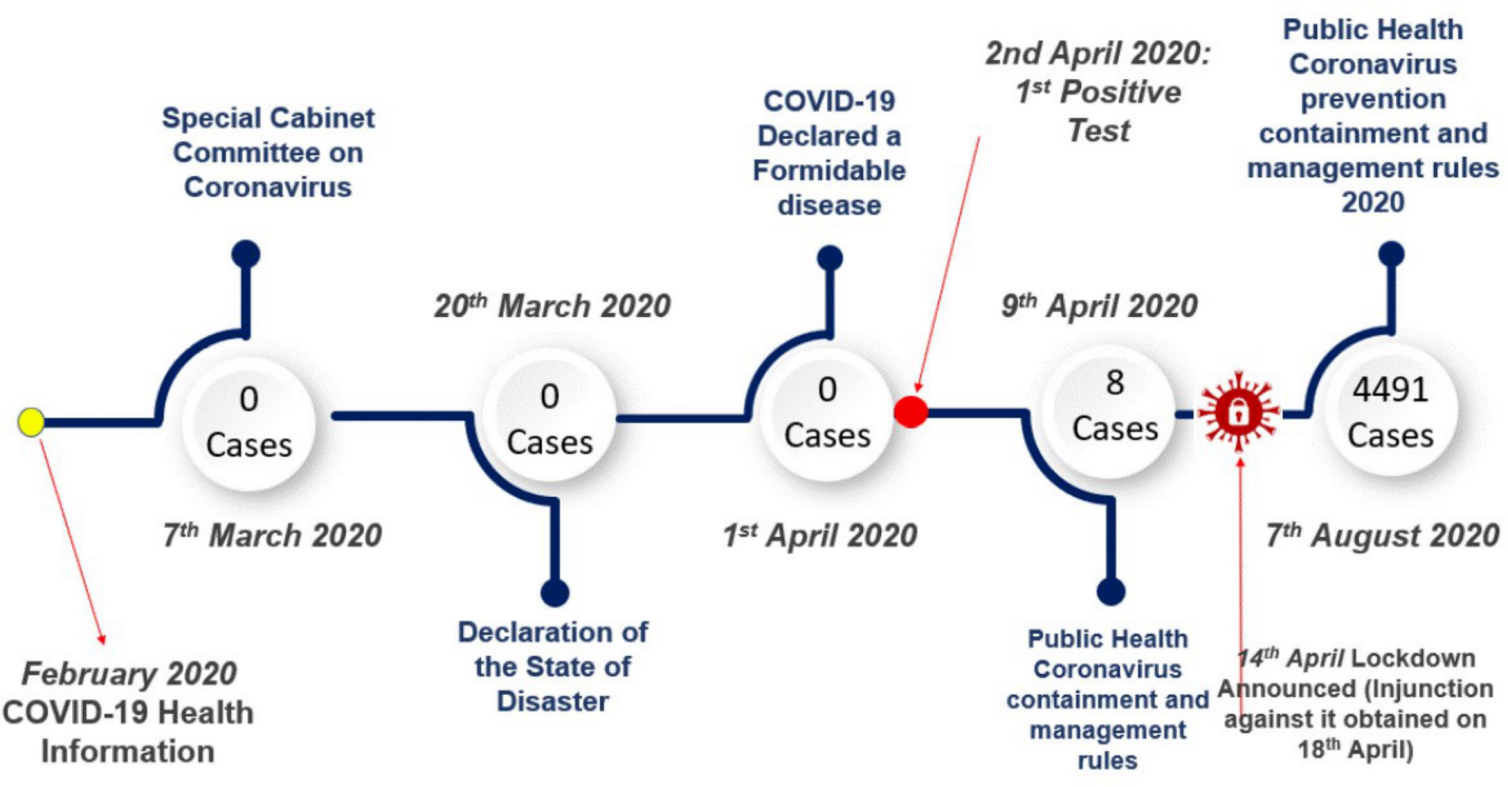
Timeline for COVID-19 pandemic response in Malawi.

The president of Malawi also created a multidisciplinary Special Cabinet Committee on Coronavirus on 7 March (figure 1).^19^ The committee had a leadership role in formulating and overseeing the implementation of key policy decisions.

#### Risk communication

The Government of Malawi health information campaign began before the first case of COVID-19 in Malawi. On the 11 February, the Government issued a statement of caution (citizens being encouraged to avoid high risk areas), and a coordinated public information campaign began on the 7 March 2020 (figure 1).^16^ According to the PHIM, COVID-19 health information campaigns included radio, television and social media messaging in English, Chichewa and Tumbuka,^12^ the three most widely spoken languages in Malawi. A mobile phone messaging system, ‘Chipatala Chapa Foni’ (Hospital on the Phone)—adapted from a Village Reach-supported M-health programme which commenced in 2011—and a toll-free number 54747, were used to allow the public to gain information about possible COVID-19 symptoms from health authorities.^20 21^

#### Policies to reduce infection transmission

A state of national disaster was declared on the 20 March 2020 and COVID-19 was declared a formidable disease on the April 2020.^22 23^ Containment policies, which are policies intended to reduce disease transmission through reduction of human-to-human contact, were implemented in several ways. Nationwide, schools across all levels were to close on the 23 March (table 1). Schools remained closed for 5 months and were announced to reopen on the 7 September 2020.^24^ There was an immediate ban on all public events and gatherings were restricted to less than 100 people. This ban was intended for all religious gatherings, weddings and funerals.^25^

**Table 1:** Major policies in response to COVID-19 in 2020

Date policy was in effect	Actor	Policy or policy action	No of confirmed COVID-19 cases
7 March	President	Creating the Presidential Task force on COVID-19.^25^	0
20 March	President	Declaration of the state of disaster and restriction of public gatherings and public events.^22^	0
23 March	President	School closures at all levels.^25^	0
24 March	MOH	Self-quarantine for individuals returning from high-risk countries (Malawi Ministry of Health Announcement for the 24 of March 2020)	0
1 April	MOH	COVID-19 disease declared a formidable disease.^23^	0
1 April	MOH	Border closure and suspension of international flights.^25^	0
9 April	MOH	Public health (coronavirus prevention, containment and management) rules, 2020.^27^	8
7 August	MOH	Public health (coronavirus prevention, containment and management) rules, 2020.^29^	4491

MOH, Malawi Ministry of Health.

On 4 April, the Government of Malawi recommended the closing of workplaces for non-essential staff.^26^ Although public transport was not closed, it was recommended to reduce the seating capacity by 60%.^25^ There was no curfew implemented for people to be at home although opening hours were reduced for restaurants. There were no restrictions on internal movement and no policies that were limited to only one geographical region in the country at any point during this period.

All commercial flights and entry into Malawi by foreign nationals were suspended but land borders remained open for cargo and returning residents who were required to undergo 14 days of quarantine.^25^

The COVID-19 response strategy involved creating the public health (corona virus prevention, containment and management) rules to clarify the roles of public health authorities and the restrictions they could impose in implementing COVID-19 response policies.^27^ These were published in the Malawi gazette on the 9 April 2020.^27^

On 14 April, the President of Malawi announced a national lockdown to begin on the 18 April for 21 days.^28^ An injunction was obtained against this lockdown on the 18 April and an amendment to the Coronavirus prevention rules was published in the Malawi gazette on the 7 August 2020.^29^ The lockdown policy was subsequently overruled on 10 September by the Malawi High Court.^28^ The judgement ruled that the restrictions suggested during the lockdown would be unconstitutional because of their negative consequences on the socioeconomic state of the majority of Malawians.^28^

#### Face coverings

The Malawi MOH recommended and encouraged use of face masks in public on 30 April.^30^ Wearing face masks (cloth or medical) in public became mandatory nationwide after MOH published the regulation in the national gazette on 7 of August.^29^

#### COVID-19 testing policy

The capacity for COVID-19 testing increased between March to August 2020 and represented a significant investment in infrastructure and equipment for health. In early March there was one COVID-19 testing centre, the National Health Reference Laboratory; by the end of March there were three, including the University of Malawi College of Medicine and the Malawi-Liverpool Wellcome Trust.^25 31^ By 31 August, 15 laboratories were using Abbott reverse transcriptase PCR tests for COVID-19 and 37 laboratories were using GeneXpert for testing in Malawi (online supplemental file).^31^ In total, 45 505 tests, including 6089 contact tracing tests, were conducted by testing sites in all districts in the country by the 31 August 2020.^31^

10.1136/bmjgh-2021-006035.supp1 Supplementary data

According to the PHIM announcement on 9 March, the criteria for COVID-19 testing was suspected cases or close contacts and symptomatic returning travellers’ residents. However, COVID-19 testing was intermittently interrupted as on the 10 July, the presidential task force on COVID-19 reported a shortage in test kits due to logistical problems in their delivery.^32^

Despite increasing the capacity for COVID-19 testing, seroprevalence studies suggested higher rates of infection than were reported from confirmed cases. Data from a study in one urban Malawian city suggested high local transmission of COVID-19 and found a seroprevalence of 12.3% of healthcare workers.^33^ This makes it difficult to understand the impact response policies had on managing the pandemic.^33^

#### Socioeconomic response policies

The economic response strategy was aimed at supporting livelihoods and was supported by development partners. The Government of Malawi expanded the existing Malawi National Social Cash Transfer Programme to the Government Urban Cash Initiative in order to reach urban areas.^18^ The programme provided unconditional cash transfers to 185 000 poor households in rural and urban areas and amounted to 0.6% of Malawi’s Gross Domestic Product.^18 34^ Other policies included tax waivers, credit facilities for small and medium enterprises and increasing the health workforce by hiring 2000 healthcare workers.^34^

### The actors involved in policy development

The process of policy formulation in response to the COVID-19 pandemic suggests that the policies were formed by national actors and development partners. In Malawi, the main leading actors were the President of Malawi and the MOH, guided by experts in healthcare, public health, epidemiology and economics in the cluster leads and the Presidential Taskforce on COVID-19. The third and fourth crucial actors in the COVID-19 policy formulation process in Malawi were the people, that is, Malawians and the judiciary. These actors influenced how policies were made and how they would be adopted.

### The context of COVID-19 policy development process

The context in which the policy formulation process occurred in Malawi was affected by the political climate of the time. In Malawi, April 2020 was 2 months since the High Court ordered fresh presidential elections—challenging the incumbent government—after the May 2019 presidential elections were nullified.^35^ The COVID-19 response policies restricted gatherings like weddings and funerals, but political rallies occurred that did not adhere to these measures.^35^ This created an atmosphere of public mistrust in the government and negatively affected public perception of COVID-19 prevention measures.^35^ It is plausible that this would affect public compliance to prevention measures.

### The process of developing health response policies

Multiple national and international actors influenced the process of policy development during the COVID-19 pandemic in Malawi. Although we did not find documents to explicitly state how specific health problems were identified, we can infer that WHO guidance and early mathematical modelling for Malawi helped to direct which policies were formed. With this information, the presidential committee on COVID-19 was mainly responsible for formulating policies. The various clusters were responsible for communicating and implementing policies and were coordinated by the National Disaster Preparedness and Relief Committee. The clusters also monitored their performance using a monitoring matrix based on their specified goals.^18^

One important event during the adoption of COVID-19 policies in Malawi was the public resistance to the diffusion of the lockdown policy. Policy diffusion is the process where a government implements a policy through the direct or indirect influence of another country or international authority.^36^ During a pandemic, low income countries could implement policies similar to high-income countries due to this process.^37^

Between March and April 2020, seven out of the nine African countries studied by Haider *et al*, implemented lockdowns involving home confinement.^38^ In Malawi, after eight confirmed cases of COVID-19, the Minister of Health announced a 21 day nationwide lockdown.^23^ Nationwide demonstrations ensued and an injunction against it was granted on the 18^th^ of April 2020 in the case of: Kathumba and others vs the President of Malawi and others.^28 39^ Although this may reflect the context of public mistrust, it also demonstrated differing interests between the government and the public at that time. A survey by the institute of public opinion and research in May 2020, revealed that 81% of Malawians feared going hungry during the COVID-19 pandemic more than they feared being infected by the virus itself.^40^ Furthermore, the majority of Malawians depend on agriculture (59% of women and 44% of men) and unskilled manual labour (20% of women and 25% of men) for work.^41^ Therefore, policies that reduce the ability to sustain livelihoods may be challenging to adopt. This resistance to the lockdown policy, reinforces the importance of the people at the centre of any health system, and as important actors in health policy.

### Unintended consequences of COVID-19 response policies

Despite the benefit of preventing the spread of the disease, response policies to contain the virus had unintended consequences. Firstly, the 5 month long school closures could increase school dropouts, teenage pregnancies and worsen social determinants of health in Malawi as was reported in many parts of sub-Saharan Africa.^42^ Indeed, Malawi registered an increase in teenage pregnancies and an 11%–99% increase in child marriages between May and July 2020 when compared with 2019 within the same time frame.^43^ Because years of schooling is a major contributor to multidimensional poverty in Malawi, deliberate efforts are needed to counter the social impacts of the pandemic for this vulnerable group.^8^ On a positive note, the Department of Education launched an electronic based learning platform for primary, secondary and tertiary school levels.

Second, while only 3% of children who met the COVID-19 case definition tested positive for the virus, there was a decrease in total paediatric hospital attendances at a tertiary hospital in Malawi during 2020 when compared with the year 2019.^44^ There was an increase in the number of children pronounced dead on arrival and a rise in children reporting sexual assault at times coinciding with school closures.^44^ Negative unintended consequences to population health have also been identified through reduced case-detection of malaria, reduced uptake of isoniazid preventive therapy, increase in maternal mortality and disruptions health seeking behaviour in Uganda.^45^ This being a new pandemic we hypothesise that the unintended consequences of the response policies are broad and likely to have long-term effects. Studies are needed to uncover them to create policies that address their possible harm to the population.

### Actions to strengthen the health system

Further research should include a comprehensive evaluation of the pandemic preparedness and response plan to understand the outcomes of these activities and how to improve on them. Qualitative research is needed to understand the public perception of health information and the factors that affect public compliance to health policies to guide health policy formulation processes.

We recommend that the public health act of 1948 be revised to accommodate pandemic response frameworks and new public health policies.^46^ This recommendation is supported by the Malawi High Court ruling on the 10th of September 2020 where the court announced that the current public health act would make it difficult to lawfully implement COVID-19 response policies.^28^ A revised act would reflect current evidence for epidemic control and become the basis for evidence-based epidemic response policies.^47^

## Conclusion

The Government of Malawi employed a multisectoral approach to managing the COVID-19 pandemic. The response, in a least developed country with limited resources and infrastructure, nevertheless targeted health, economic and social health aspects of the COVID-19 pandemic. We highlight the need for public engagement to facilitate public compliance to preventative measures, and to revise the public health act of Malawi to support epidemic response policies. Further research is needed to understand the impact of response policies and the factors that affect the compliance of the public to epidemic response policies.

A major limitation to our document review was that there were few official documents that clearly state the policies implemented and what analyses, if any led to those policies. Policies were mainly announced by policy actors in authority and there were few published policy documents available on government websites. Lastly, there was lack of clear documentation on how policies were identified and the evaluation of their implementation or impact have not been published.

## Data Availability

Data sharing not applicable as no datasets were generated and/or analysed for this study.
