# Supplementary material for: The health policy response to COVID-19 in Malawi
Source: BMJ Glob Health. 2021 May 18;6(5):e006035. doi: 10.1136/bmjgh-2021-006035 (PMC8136801; doi:10.1136/bmjgh-2021-006035)
Supplement: Supplementary data [file bmjgh-2021-006035supp001.pdf]

Table of COVID-19 Laboratory Testing Sites in Malawi on 31st August 2020

Region	District	Testing Sites
Southern Region	Blantyre	Blantyre Dream
		Blantyre Light House
		College of Medicine
		Malamulo Adventist
		Malawi Liverpool Wellcome Trust
		Queen Elizabeth Central Hospital
	Mwanza	Mwanza District Hospital
	Mangochi	Mangochi District Hospital
	Zomba	Zomba Central Hospital
	Balaka	Balaka Dream
		Balaka District Hospital
	Thyolo	Thyolo District Hospital
	Neno	Neno District Hospital
	Phalombe	Phalombe District Hospital
	Chiradzulu	Chiradzulu District Hospital
	Mulanje	Mulanje District Hospital
	Chikwawa	Chikwawa District Hospital
	Nsanje	Nsanje District Hospital
	Machinga	Machinga District Hospital
Central Region	Lilongwe	Bwaila Hospital
		Kamuzu Central Hospital
		Kamuzu Barracks
		Lilongwe Light House
		Partners in Health
		UNC Project Lilongwe
	Salima	Salima District Hospital
	Mchinji	Mchinji District Hospital
	Dowa	Dowa District Hospital
	Kasungu	Kasungu District Hospital
	Nkhotakota	Nkhotakota District Hospital
	Dedza	Dedza District Hospital
		Nkhoma Mission
	Ntcheu	Ntcheu District Hospital
	Ntchisi	Ntchisi District Hospital
Northern Region	Mzimba	Mzuzu Central Hospital
		Mzimba South District Hospital
		Wezi Medical Centre
	Nkhata Bay	Nkhata Bay District Hospital
	Karonga	Karonga District Hospital
		Karonga MEIRU
	Chitipa	Chitipa District Hospital
	Likoma	Likoma
	Rumphi	Rumphi District Hospital

MEIRU: Malawi Epidemiology and Intervention Research Unit
